# Screening of Rhizospheric Actinomycetes for Various *In-vitro* and *In-vivo* Plant Growth Promoting (PGP) Traits and for Agroactive Compounds

**DOI:** 10.3389/fmicb.2016.01334

**Published:** 2016-08-29

**Authors:** Sumaira Anwar, Basharat Ali, Imran Sajid

**Affiliations:** Department of Microbiology and Molecular Genetics, University of the PunjabLahore, Pakistan

**Keywords:** plant growth promoting *Streptomyces*, indole acetic acid (IAA), wheat, 16S rRNA gene sequencing, biofertilizers, agro-active compounds

## Abstract

In this study 98 rhizospheric actinomycetes were isolated from different wheat and tomato fields, Punjab, Pakistan. The isolates were characterized morphologically, biochemically, and genetically and were subjected to a comprehensive *in vitro* screening for various plant growth promoting (PGP) traits. About 30% of the isolates screened were found to be the promising PGP rhizobacteria (PGPRs), which exhibited maximum genetic similarity (up to 98–99%) with different species of the genus *Streptomyces* by using16S rRNA gene sequencing. The most active indole acetic acid (IAA) producer *Streptomyces nobilis* WA-3, *Streptomyces Kunmingenesis* WC-3, and *Streptomyces enissocaesilis* TA-3 produce 79.5, 79.23, and 69.26 μg/ml IAA respectively at 500 μg/ml L-tryptophan. The highest concentration of soluble phosphate was produced by *Streptomyces* sp. WA-1 (72.13 mg/100 ml) and *S. djakartensis* TB-4 (70.36 mg/100 ml). All rhizobacterial isolates were positive for siderophore, ammonia, and hydrogen cyanide production. Strain *S. mutabilis* WD-3 showed highest concentration of ACC-deaminase (1.9 mmol /l). For *in-vivo* screening, seed germination, and plant growth experiment were conducted by inoculating wheat (*Triticum aestivum*) seeds with the six selected isolates. Significant increases in shoot length was observed with *S. nobilis* WA-3 (65%), increased root length was recorded in case of *S. nobilis* WA-3 (81%) as compared to water treated control plants. Maximum increases in plant fresh weight were recorded with *S. nobilis* WA-3 (84%), increased plant dry weight was recorded in case of *S. nobilis* WA-3 (85%) as compared to water treated control plants. In case of number of leaves, significant increase was recorded with *S. nobilis* WA-3 (27%) and significant increase in case of number of roots were recorded in case of strain *S. nobilis* WA-3 (30%) as compared to control plants. Over all the study revealed that these rhizospheric PGP *Streptomyces* are good candidates to be developed as bioferlizers for growth promotion and yield enhancement in wheat crop and can be exploited for the commercial production of different agro-active compounds.

## Introduction

Effective farming practices, now-a-days, rely on extensive use of chemical fertilizers in order to enhance plant growth and yield. However, the cost, environmental concerns and the resulting human health hazards due to the inclusion of these chemical fertilizers in food chain are the major limiting factors. Microorganisms have been considered an important source of natural compounds of agro active importance. Use of microbial consortia in the form of bio fertilizers for reduction in the application of chemical fertilizers, pesticides and related agrochemicals, without compromising the plant yield is currently a significant research area in the field of agriculture, microbiology, and biotechnology (Ahmad et al., 2008).

Plant growth promoting rhizobacteria (PGPR) is a group of naturally occurring, free living rhizosphere colonizing bacteria that improve plant growth, increase yield, enhance soil fertility, and reduce pathogens as well as biotic or abiotic stresses (Vessey, 2003; Kumar et al., 2014). PGPR help the plants by producing plant growth phytohormones such as indole acetic acid (IAA), cytokinins, and gibberellins (Marques et al., 2010), solubilization of inorganic phosphate (Jeon et al., 2003), asymbiotic nitrogen fixation (Khan, 2005), antagonistic effect against phytopathogenic microorganisms by producing siderophore, antibiotics, and fungicidal compounds (Lucy et al., 2004; Barriuso et al., 2008; Majeed et al., 2015).

Actinomycetes are present extensively in the plant rhizosphere and produce various agroactive compounds. In the last few years, this group of bacteria, due to its strong antimicrobial potential, and soil dominant saprophytic nature, gained much attention as plant growth promoters (PGP; Franco-Correa et al., 2010). Actinobacteria can actively colonize plant root systems, can degrade a wide range of biopolymers by secreting several hydrolytic enzymes and tolerate hostile conditions by forming spores (Alexander, 1977). Actinobacteria, especially *Streptomyces*, also exhibit immense biocontrol action against a range of phytopathogens (Wang et al., 2013). Actinobacteria can produce phytohormones (IAA) and siderophore as well as solubilize phosphate and promote plant growth (Jeon et al., 2003). Actinomycetes have been mainly exploited in pharmaceutical industry since 1940s, Whereas, only a few have been developed as commercial products for plant application in agriculture (Minuto et al., 2006). Streptomycetes have been long considered simply as free-living soil inhabitants, but recently the importance of their complex interactions with plants, and other organisms is being uncovered (Seipke et al., 2011).

Interest in the beneficial rhizobacteria associated with cereals has increased recently and several studies clearly demonstrated the positive and beneficial effects of PGPR on growth and yield of different crops especially wheat at different environments under variable ecological conditions (Marques et al., 2010; Mehnaz et al., 2010; Zhang et al., 2012). Wheat (*Triticum aestivum*) ranked third most abundant cereal crop after maize and rice and covers approximately 30% of the total cereal products worldwide (Fageria and Baligar, 1997). Pakistan is the 18 largest wheat producing country in the world (Tunio et al., 2006) with an average of 20–24 million tons of wheat grown a year. Wheat is grown on major areas in Pakistan but its average yield per hectare is far less than the actual potential (Akhtar et al., 2009). Understanding the diversity and distribution of indigenous actinobacteria in the rhizosphere of particular crops is depended on the knowledge of native actinobacterial populations, their isolation, identification, and characterization. It is therefore mandatory to explore region specific actinobacterial strains that can be used as growth promoters to achieve desired crop production (Deepa et al., 2010). However, in spite of actinobacterial high soil population, secondary metabolite production and capability to endure hostile environments, *Streptomyces*, and other actinobacteria are unexpectedly under explored for plant-growth promotion, as compared to *Pseudomonas* or *Bacillus* spp. (Doumbou et al., 2002).

The main objectives of the present study included: to isolate indigenous actinobacteria from the wheat (*Triticum aestivum*) and tomato (*Solanum lycopersicum*) rhizosphere, characterize these isolates on the basis of morphological, and physiological characteristics as well as by 16S rRNA gene sequence analysis, to screen actinobacteria for various plant growth promoting activities (PGPAs), such as IAA production, phosphate solubilization, siderophore production, and *in-vitro* 1-aminocyclopropane-1-carboxylate (ACC) deaminase activity. The PGP potential of selected isolates was also studied *in-vivo* under axenic conditions and their effect on wheat growth was investigated. Keeping in mind, there is a lack of data on the Plant growth promotion (PGP) potential of actinomycetes isolated from Pakistan, Therefore, this study provides new and novel information.

## Materials and methods

### Collection and enrichment of soil samples

The soil samples for the isolation of actinobacteria were collected from the rhizosphere of wheat (*Triticum aestivum*) and tomato (*Solanum lycopersicum*) plants cultivated in the Punjab province (District: Lahore, Gujranwala, Sheikhupura) Pakistan, during the months of December, 2013–March, 2014. Samples were collected in properly labeled sterile polythene sampling bags. The samples were subjected to physical treatment (heating, 55°C/60 min) according to the method of Seong et al. (2001) and chemical treatment; CaCO_3_: soil (1:10 w/w; Oskay, 2009), for the enrichment of actinobateria.

### Isolation and preservation of actinobacteria

One Gram of soil sample was mixed in 9 ml of autoclaved water and serially diluted to a final dilution of 10^−3^, 10^−4^, and 10^−5^. The 0.1 ml of each dilution was spreaded on ISP-4 medium (Soluble starch 10 g, CaCO_3_ 2 g, (NH_4_)_2_SO_4_ 2 g, K_2_HPO_4_ 1.0 g, MgSO_4_.7H_2_O 1.0 g, NaCl 1.0 g, FeSO_4_.7H_2_O 1.0 mg, MnCl_2_.7H_2_O 1.0 mg, ZnSO_4_.7H_2_O 1.0 mg, Agar 15 g, distilled water 1 L, pH = 7.5 ± 0.2, 28°C) supplemented with 25 μg/ml nalidixic acid and 50 g/ml nystatin as antibacterial and antifungal agents (Taechowisan et al., 2003). Isolated actinobacteria were sub-cultured on ISP-2 medium (Yeast extract 4 g, Glucose 4 g, Malt extract 10 g, Agar15 g, distilled water 1 L, pH = 7.5) and the plates were incubated at 28°C for 7–12 days. All the isolated strains were preserved in 25% glycerol and kept at −80°C. After incubation, 98 colonies of actinobacteria were selected on the basis of morphological characters such as distinct color and colony shape. Among them, 30 strains were detected to be exhibiting Plant growth promoting activities in initial *in-vitro* screening. Finally, six isolates that included WA-1, WA-3, WC-3, WD-3, TA-3, and TB-4 which exhibited most significant PGP traits were selected.

### Taxonomic studies

#### Biochemical and physiological characterization

A comprehensive morphological, biochemical, and physiological characterization scheme was adopted to determine the taxonomic status of the selected actinobacterial strains. Among the different biochemical characteristics studied, melanin pigment production was determined by following the methods described by Pridham and Lyons (1969) and positive results were indicated by blackening of the media. Carbohydrate utilization was performed by using ISP-9 medium supplemented with different sugars. Decomposition of oxalic acid and other organic acids was performed by using the method of Nitsch and Kutzner (1969). Esculin degradation was detected by blackening of the media after 12 days of incubation was recorded as positive result. Trysoine agar was used for determining tyrosine hydrolysis by actinomycetes (Gordon and Smith, 1953). Xanthine and hypoxanthine utilization is determined by clear zone formation around the colony after 12 days of incubation (Gordon and Smith, 1953). Starch hydrolysis was performed as described by Cowan (1974). After 12 days of incubation, plates were flooded with lugol's iodine and clear zone around colonies were recorded as positive. Urease releases ammonia from urea and the increase in the pH was detected by the indicator phenol red changing from yellow to pink (Gordon et al., 1974), Cell wall type was determined based on the isomers of diaminopimelic acid (DAP) by using the method of Becker et al. (1964). Cultural characteristics such as color of aerial and substrate mycelium and pigmentation of the selected actinobacteria was recorded on ISP-2 medium according to the method of Shirling and Gottlieb (1966). Growth at different pH 5, 6, 7, 8 was noted after 14 days of incubation on the agar plates. Tolerance to temperature was tested at 10, 28, 37, 45°C and visible growth was recorded as positive result. Agar supplemented with different NaCl Concentration 0, 4, 7, 10, 13% was used for determining NaCl tolerance of actinomycetes.

### *In-vitro* screening of actinobacteria for their plant growth promoting activities

#### Colorimetric analysis of indole acetic acid (IAA) production

Auxin form different strains of actinomycetes was quantified using the method of Tang and Borner (1979) as described by Ali et al. (2009). All Actinobacteria were grown at 28°C in ISP-2 liquid medium in triplicates for 7–12 days at 120 rpm on an orbital shaker. Media treatments were supplemented with different concentrations of L-tryptophan (0, 100, 200, 300, 400, and 500 μg/ml). Cells were removed from culture medium by centrifugation at 14,000 rpm for 15 min (Sigma 2–5, sigma Laborzentrifugen, Osterode, Germany). The supernatant (1 ml) was mixed with 2 ml of Salkowski's reagent (50 ml, 35% perchloric acid, 1 ml of 0.5 M FeCl_3_ solution) and was incubated at room temperature for 30 min in dark. Development of pink or red color indicates IAA production. Optical density was taken at 535 nm by using spectrophotometer (S-300D; R and M Marketing, Hounslow, UK). Standard curve of IAA was used to measure the concentration of IAA produced by the actinobacteria.

### Siderophore production

The strains were assayed for the siderophore production on the Chrome Azurol S agar according to the method of Alexander and Zuberer (1991). CAS agar plates were prepared and spot inoculated with actinobacterial strains and incubated at 28°C for 7 days. The colonies producing yellow to orange halos were considered positive for siderophore production.

### Solubilization of phosphate

Pikovskaya's agar plates were used for qualitative screening of all actinobacterial isolates (Gaur, 1990). Solubilization index (SI) was calculated by using the formula of Edi Premono et al. (1996). King (1932) method was used for quantitative analysis of solubilization of tri-calcium phosphate in liquid medium.

### Ammonia production

Freshly grown actinobacterial cultures were inoculated into 1 ml of peptone water and incubated at 28°C for 7–12 days with shaking at 120 rpm. After incubation, 0.5 ml of Nessler's reagent was added in each culture tube. Development of yellow to brown color indicates positive result for ammonia production (Cappuccino and Sherman, 2002).

### Hydrogen cyanide production

Producton of hydrogen cyanide by actinobacterial culture was evaluated by adapting the method of Lorck (1948). Actionobacteria were streaked on ISP-2 medium amended with 4.4 glycine/l and whatman filter paper No. 1 dipped in 2% sodium carbonate in 0.5% picric acid for a minute was placed underneath the petri plates lids. Plates were sealed with parafilm and incubated at 28°C for 7–12 days. Orange to red color of filter was indicative of HCN production.

### Colorimetric ninhydrin assay for screening ACC utilizing actinobacteria

Li et al. (2011) method was used. ISP-2 medium was inoculated with actinobacteria, incubated at 28°C with shaking 200 rpm for 7–12 days. After centrifugation of 2 ml of culture at 14,000 rpm for 5 min, supernatant was discarded, pellet washed with 1 ml of liquid DF- medium, later mixed with 2 ml of ACC substrate containing DF mineral medium. 2 ml of DF-ACC medium without inoculation served as control. All the samples were incubated for 4 days at 200 rpm. After incubation, 1 ml of each bacterial culture was centrifuged at 14,000 rpm for 5 min. 100 μl of each supernatant was shifted to another tube and was diluted with 1 ml of liquid DF medium. In 96-well PCR plate, 60 μl of each diluted supernatant was mixed 120 μl of ninhydrin reagent, covered with parafilm, and placed in boiling water bath for 30 min. DF medium was used as a blank.

The resulted purple color depth was record for visual comparison of actinobacterial strains. 100 μl of the reaction mixture was transfer to the microtitre plate in triplicates and absorbance was taken at 570 nm with spectrophotometer. The actinobacterial isolate with visibly reduced color depth and less supernatant absorbance compared to DF-ACC medium without inoculation were considered as ACC utilizing actinobacterial strains.

### *In-vivo* screening for plant growth promotion activities

Actinobacterial isolates were grown on ISP-2 broth at 28°C for 7–12 days with continuous shaking 200 rpm. Actinobacterial suspensions were prepared according to the method of Errakhi et al. (2007). Wheat seeds were surface sterilized by using the method of Khalid et al. (2004). Sterilized seeds were soaked for 1 h in the suspension and dried under laminar flow hood overnight. For control, seeds were dipped in distilled water only.

### Germination bioassay

Germination assay was performed by using actinobacterial treated wheat (*Triticum aestivum*) seeds (three replicates, 5 seeds/plate) were placed in sterilized petri dishes covered with two sheets of filter papers, moistened with 10 ml of sterile distilled water. Water treated control seeds were used. All the petri dishes were incubated in Versatile Environmental Test Chamber (TEMI 850 WISE CUBE) with light intensity of 2000 lux for 16 h daily at 28°C. After 2 weeks, effect of actinobacterial cultures on root length and number of roots was observed.

### Pot experiment

Actinobacterial treated eight wheat seeds (as described above) were sown to a depth of 1 cm in plastic pots (12 cm high × 10 cm diameter) filled with sterilized soil. Three replicates were used for actino-bacterial as well as for control treatments. For control, seeds were dipped in distilled water only. Pots were arranged in fully randomized complete block, under standard conditions, in plant growth room (22–26°C, 16 h light/8 h dark). All pots were watered daily with 10 ml of distilled autoclaved water to achieve moisture level sufficient for seed germination. After 25 days, five plants were removed carefully from the soil, washed with tap water to remove soil particles. Total 15 plants per treatment were considered for statistical analysis. Data was recorded for number of seeds germinated, root and shoot length, root and shoot fresh weight, root, and shoot dry weight (80°C, 24 h electric oven) and number of roots.

Root colonization potential of inoculated Streptomyces was determined at every 8 days by using serial dilution plating technique on GYM agar and number of viable cells was recorded as colony forming units (CFU) as described in Somasegaran and Hoben (1994).

### Genomic DNA isolation, PCR amplification and sequencing of the 16s rRNA gene

Total genomic DNA was isolated according to the CTAB method described by the Liu et al. (2000). Universal Primers 27F 5′ AGAGTTTGATCMTGGCTCAG 3 and 1492 R′ TACGGYTACCTTGTTACGACTT 3′ were used for the PCR amplification of 16S rRNA gene of the selected strains. Agarose gel electrophoresis (1%) was used for analyzing PCR product and remaining mixture was purified by using PCR Purification kit (FavorPrep™). Purified PCR products were sequenced commercially by GATC Biotech (Germany). The obtained gene sequences were compared with others in the Gen Bank databases using the NCBI Nucleotide BLAST at http://blast.ncbi.nlm.nih.gov/Blast.cgi. Sequences were submitted to NCBI GenBank data base and accession numbers were obtained.

### Statistical analysis

For all experiments, the data were subjected to statistical analysis using software IBM SPSS Statistics version 21. Data were subjected to analysis of variance (ANOVA) and means separated using Duncan's multiple range test (*P* = 0.05). The correlation coefficients between bacterial auxin production and L-tryptophan concentrations as well as between bacterial growth traits and plant growth parameters were also calculated (*P* = 0.01 or *P* = 0.05).

## Results

### Taxonomic characteristics of the selected rhizopsheric actinomycetes

Ninty eight actinomycetes were isolated from six different wheat and four different tomato rhizospheric soil samples. All of them were found to be gram positive filamentous rods. On the basis of color of aerial mycelium they were grouped into gray (TA-3, TB-4), yellow (WC-3, WD-3), green (WA-1), and orange (WA-3) color series. Diffusible pigment was produced by the isolate WC-3. All strains contained LL-diaminopimelic acid isomer in their cell wall. Physiologically, most of the actinomycetes isolates were able to utilize different sugars as the carbon source. All strains were able to utilize glucose, xylose, galactose, arabinose, mannose, and mannitol. Strain WA-1 and TA-3 were unable to utilize sucrose, while strain WC-3, and WD-3 were unable to utilize raffinose as their carbon source (Table 1). Most of them were able to grow at temperature 28 and 37°C. None of them were able to grow at temperature 4°C. Optimum temperature and pH was found to be 28°C and eight respectively (Table 2; Tables S1, S2, Figures S1, S2).

**Table 1:** **Morphological, biochemical, and physiological characteristics of the selected rhizospheric actinomycete strains**.

**Characteristics**	**Actinomycete isolates**					
	**WA-1**	**WA-3**	**WC-3**	**WD-3**	**TA-3**	**TB-4**
Colony diameter (cm)	0.6	0.3	0.3	0.4	0.5	0.2
Color of aerial mycelium	Green	Orange	Light yellow	Pale yellow	Platinum gray	Dusty gray
Color of substrate mycelium	Beige yellow	Orange red	Olive yellow	Light brown	Clay brown	Clay brown
Diffusible pigment	−	−	Beige	−	−	−
Melanoid pigment	Black	Black	−	Black	−	Black
**CARBON SOURCE UTILIZATION**						
Glucose	+	+	+	+	+	+
Xylose	−	−	−	+	+	+
Galactose	+	+	+	+	+	+
Sucrose	−	+	+	+	−	+
Mannose	+	+	+	+	+	+
Arabinose	+	+	−	+	+	−
Mannitol	+	+	+	+	+	+
Raffinose	+	+	−	−	+	+
**HYDROLYSIS OF**						
Tyrosine	+	+	−	+	−	+
Xanthine	+	−	+	+	−	+
Hypoxanthine	+	+	+	+	−	+
Starch	+	+	+	+	+	+
Urea	+	+	+	+	+	+
Decomposition of oxalic acid	+	−	−	+	+	+
Degradation of esculin	+	−	−	+	−	+

The symbol, + representing the positive reaction/presence of growth while symbol, − represents the negative reaction/absence of growth.

**Table 2:** **Temperature (°C), pH and NaCl (%) tolerance of the selected rhizospheric actinomycete strains**.

**Strains**	**Temperature tolerance**				**pH tolerance**				**NaCl tolerance**				
	**10°C**	**28°C**	**37°C**	**45°C**	**5**	**6**	**7**	**8**	**0%**	**4%**	**7%**	**10%**	**13%**
WA-1	+	+	+	+	−	−	+	+	+	+	+	+	−
WA-3	−	+	+	+	−	+	+	+	+	+	+	−	−
WC-3	−	+	+	−	−	+	+	+	+	−	−	−	−
WD-3	+	+	+	+	−	+	+	+	+	+	+	+	−
TA-3	−	+	+	−	−	−	+	+	+	+	−	−	−
TB-4	−	+	+	−	−	+	+	+	+	+	+	−	−

The symbol, + represents the positive reaction/presence of growth while symbol, − represents the negative reaction/absence of growth.

### IAA production by selected actinomycetes

**Figure 1:**
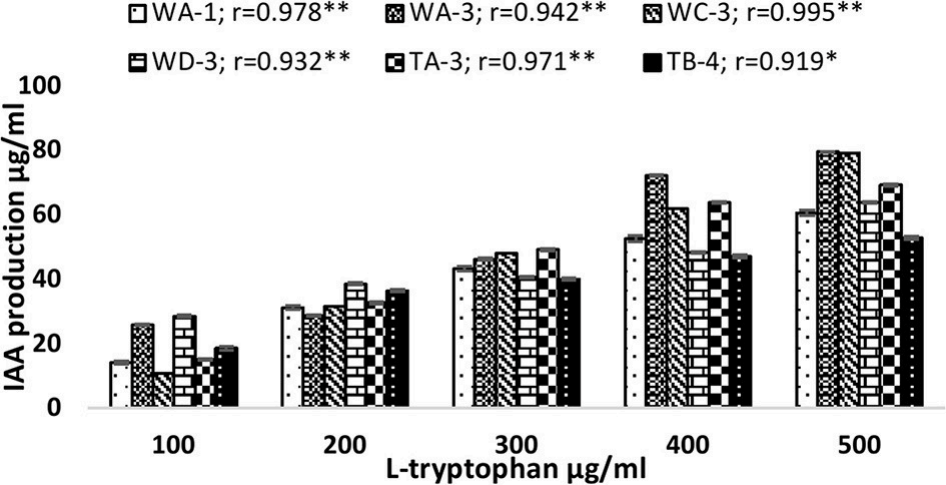
**Effect of different L-tryptophan concentrations (100, 200, 300, 400, and 500 μg/ml) on IAA production by actinomycetes**. The results shown are representative of three repetitions of the experiment. Value “r” indicates highly significant positive correlation between L-tryptophan and bacterial IAA production. **P = 0.01, *P = 0.05.

Qualitative analysis of culture supernatant of selected actinomycete isolates revealed production of variable amounts of IAA in the absence and presence of different concentrations of tryptophan (100–500 μg/ml). In the absence of L-tryptophan, IAA production was not observed. With increasing concentration of L-tryptophan, the IAA production was increased. For instance, isolate *Streptomyces* sp. WA-1(*r* = 0.979, *P* = 0.01), *S. nobilis* WA-3 (*r* = 0.942, *P* = 0.01), *S. kunmingensis* WC-3 (*r* = 0.995, *P* = 0.01), *S. mutabilis* WD-3 (*r* = 0.932, *P* = 0.01), *S. enissocaesilis* TA-3 (*r* = 0.971, *P* = 0.01), and *S. djakartensis* TB-4 (*r* = 0.919, *P* = 0.05) showed significant positive correlation with increasing L-tryptophan concentrations. Isolate WA-3 produced highest amount of IAA followed by WC-3, TA-3, WD-3, WA-1, and TB-4. The most active IAA producer *S. nobilis* WA-3, *S. Kunmingenesis* WC-3 and *S. enissocaesilis* TA-3 produce 79.5, 79.23, and 69.26 μg/ml IAA respectively at 500 μg/ml L-tryptophan (Figure 1).

### Phosphate solubilization

Among the selected isolates, six *Streptomyces* were able to solubilize phosphate by producing clear zones around the colonies after 7 days of incubation. *Streptomyces* sp. WA-1 showed highest phosphate solubilization index of 2.33 followed by *S. djakartensis* TB-4 (2.27) > *S. enissocaesilis* TA-3 (2.25) > *S. nobilis* WA-3 (2.22) > *S. mutabilis* WD-3 (2.21) > *S. kunmingensis* WC-3 (2.20). Quantitatively, the highest concentration of soluble phosphate was produced by *Streptomyces* sp. WA-1 (72.13 mg/100 ml) and *S. djakartensis* TB-4 (70.36 mg/100 ml; Table 3).

**Table 3:** **Plant growth promoting traits of the selected rhizospheric actinomycetes under *in-vitro* conditions**.

**Strains**	**Phosphate solubilization**		**ACC^b^ deaminase concentration (mmol/l)**	**Ammonia production**	**HCN^c^ production**	**Siderophore production**
	**SI^a^**	**Soluble P concentration (mg/100 ml)**				
WA-1	2.33	72.13 ± 0.336	1.65 ± 0.42 d	+	+	+
WA-3	2.22	66.0 ± 0.40	1.29 ± 0.45 b	+	+	+
WC-3	2.20	61.46 ± 0.279	1.59 ± 0.38 c	+	+	+
WD-3	2.21	57.83 ± 0.32	1.9 ± 0.35 f	+	+	+
TA-3	2.25	62.9 ± 0.40	0.71 ± 0.16 a	+	+	+
TB-4	2.27	70.36 ± 0.33	1.81 ± 0.39 e	+	+	+

a *SI, Solubilization index = the ratio of the total diameter (colony + halo zone) to the colony diameter*.

b *ACC, 1-aminocyclopropane-1-carboxylate (ACC) deaminase. Different letters on bars indicate significant difference between treatments, using Duncan's multiple range test (P = 0.05)*.

c *HCN, Hydrogen cyanide. The symbol, + represents the positive reaction/presence of trait while symbol, − represents the negative reaction/absence of trait*.

### Siderophore, ammonia, and HCN production

**Figure 2:**
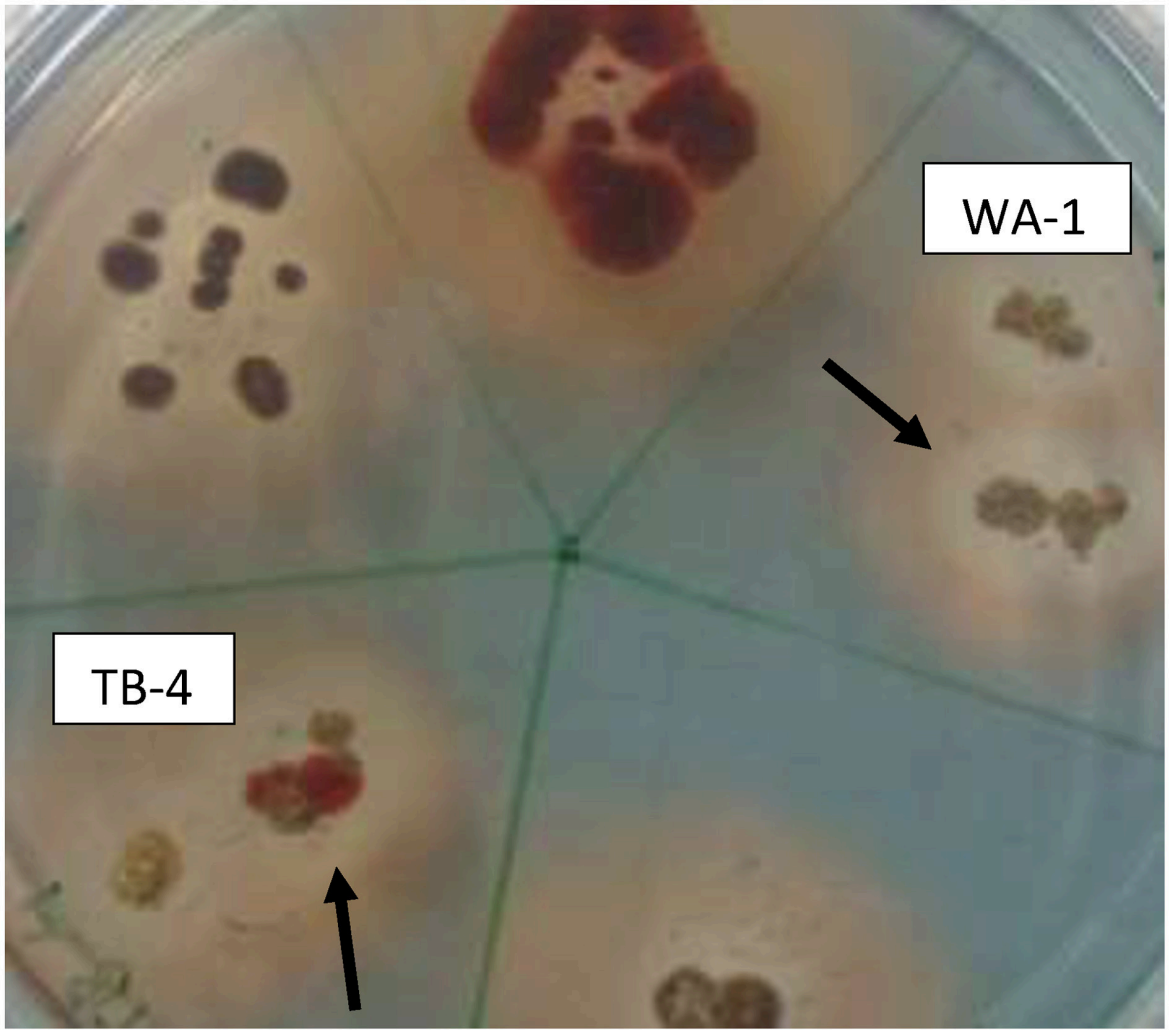
**Screening of siderophore producing actinomycete isolates using CAS agar plates after 7 days of growth at 28 ± 2°C**. The arrows indicate the halo zone around the colonies of isolate WA-1 (*Streptomyces* sp.) and TB-4 (*Streptomyces djakartensis*).

Among the selected isolates, six rhizospheric actinomycetes were positive for the ammonia production. Siderophore production was detected in all six isolates on CAS agar media, forming clear orange halo zone around the colonies. (Figure 2). All the six isolates were positive for HCN production (Table 3). *Streptomyces* sp. WA-1 and *S. djakartensis* TB-4 displayed the highest amount of HCN production as depicted by a very deep red color on the filter paper (Table 3).

### ACC deaminase activity by actinomycetes

All strains showed variable concentration of ACC-deaminase in the culture media. Strain *S. mutabilis* WD-3 showed highest concentration of ACC-deaminase 1.9 mmol /l). On the other hand, strain *S. djakartensis* TB-4, *Streptomyces* sp. WA-1, *S. kunmingensis* WC-3, *S. nobilis* WA-3 produce 1.81, 1.65, 1.59, 1.29 mmol/l concentration of ACC-deaminase, respectively. Strain *S. djarkartensis* TA-3 produce the lowest concentration (0.71) of ACC deaminase (Table 3).

### Seed germination assay

**Figure 3:**
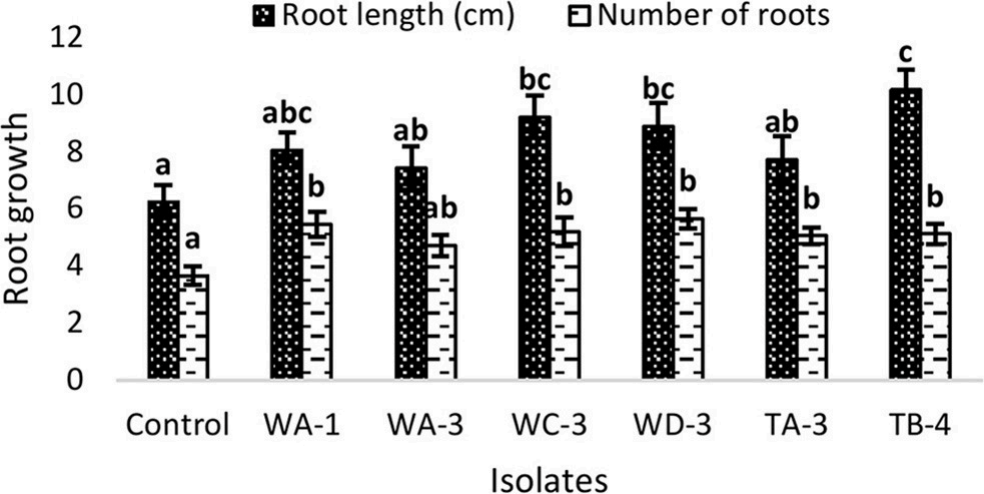
**Effect of actinomycetes spore suspension on seed germination of *Triticum aestivum***. Bars represents mean ± SE of three replicates (15 plants). Different letters on bars indicate significant difference between treatments, using Duncan's multiple range test (*P* = 0.05).

Inoculation of wheat seeds with selected actinomycetes stimulated root growth in majority of the strains (Figure 3). In case of root length, significant increases were recorded for *S. djakartensis* TB-4 (61%), *S. Kunmingenesis* WC-3 (47%) and *S. mutabilis* (42%). Increases in number of roots were also observed with *S. mutabilis* WD-3 (55%), *Streptomyces* sp. WA-1 (50%), and *S. djakartensis* TB-4 (41%).

### Root colonization potential

**Figure 4:**
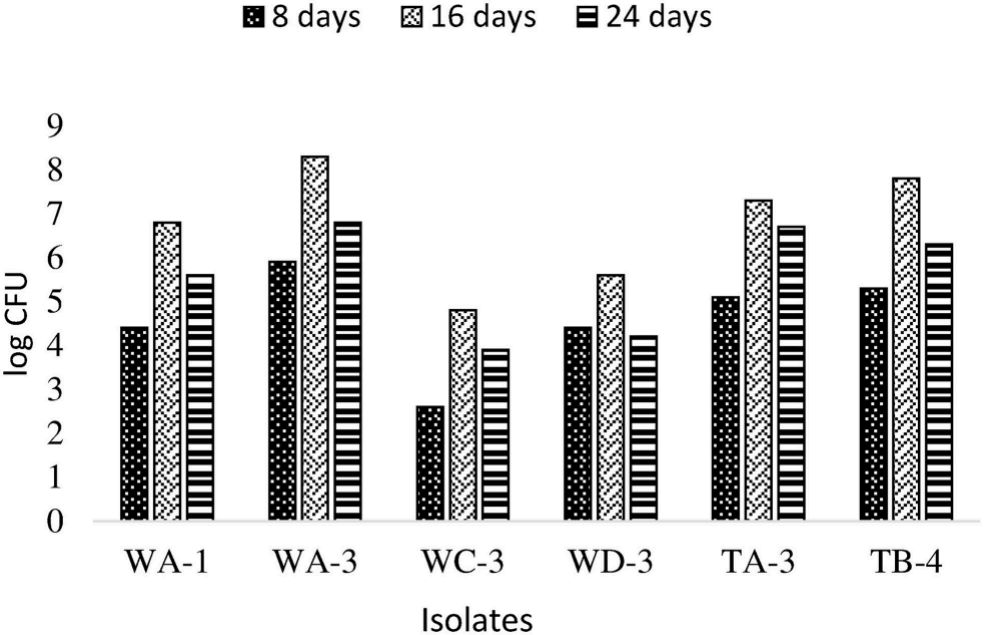
**Population density of different actinobacterial isolates inoculated to wheat at different time intervals under axenic conditions**.

Actinobacterial population size was estimated by plate count method on GYM agar at three (8, 16, 24 days) different time intervals. It was observed that all rhizospheric actinobacteria were able to colonize wheat plant roots and displayed persistence in the rhizosphere up to 25 days after inoculation (Figure 4). Maximum colonization was recorded between 8 and 16 days post inoculation. Actinobacterial isolate WA-3 showed maximum number of root colonizing colonies at all times as compared to other actinobacterial isolates.

### Plant growth promotion experiment with selected actinomycetes

**Figure 5:**
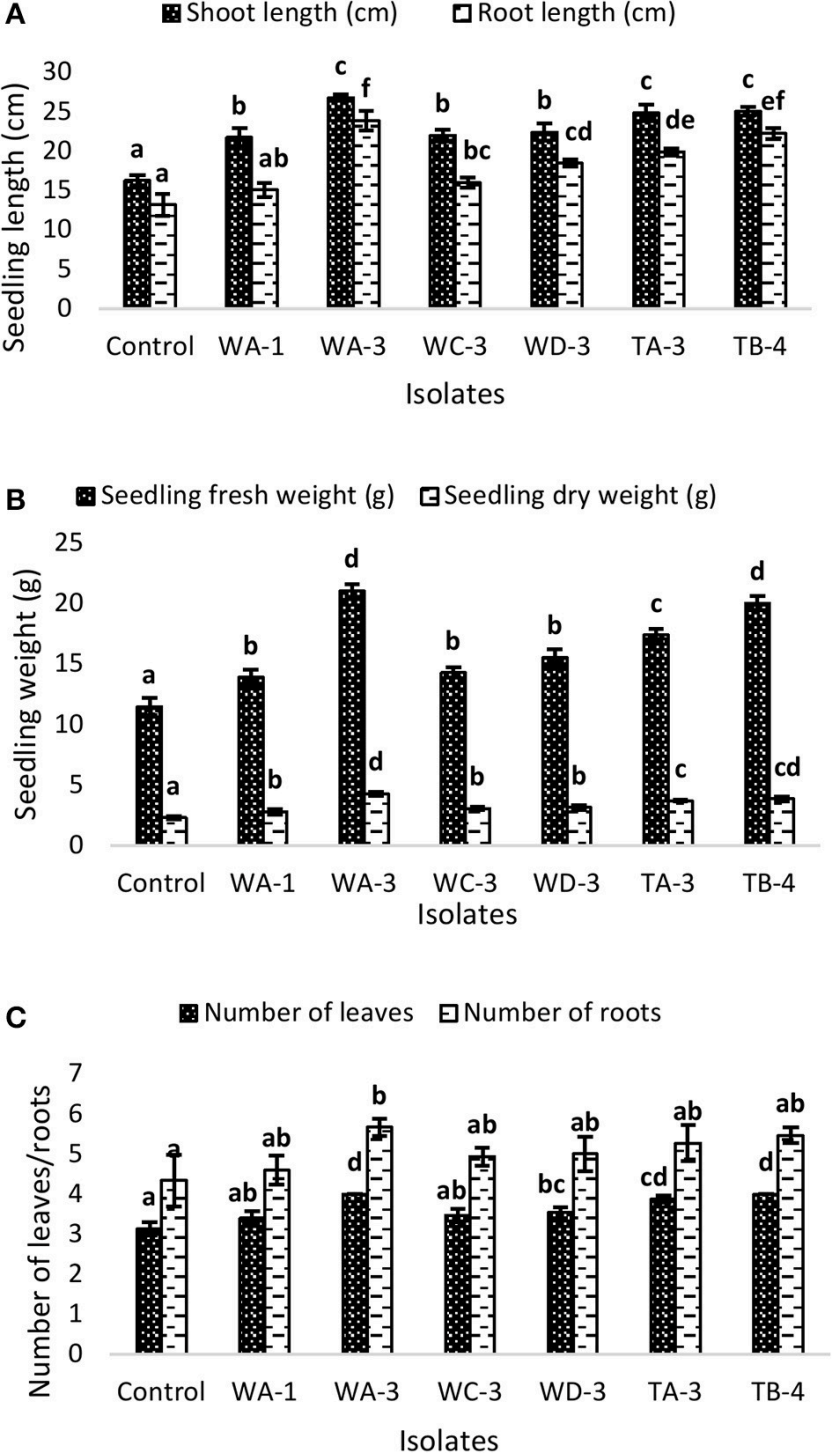
**Effect of seed treatment with actinomycetes spore suspensions on plant growth promotion of *Triticum aestivum*. (A)** Seedling root and shoot length (cm) **(B)**, seedling fresh and dry weight (g) **(C)** number of leaves and roots per plant. Evaluation was made 30 days after planting. Bars represents mean ± SE of three replicates (15 plants). Different letters on bars indicate significant difference between treatments, using Duncan's multiple range test (*P* = 0.05).

Wheat seeds inoculated with six selected actinomycetes showed variable growth and yield parameters after 3 weeks growth (Table 4). Significant increases in shoot length were observed with *S. nobilis* WA-3 (65%), *S. djakartensis* TB-4 (54%), and *S. enissocaesilis* TA-3 (53%) as compared to water treated control. Significant increase in root length was recorded with *S. nobilis* WA-3 (81%), *S. djakartensis* TB-4 (69%), and *S. enissocaesilis* TA-3 (51%) as compared to water treated control. Maximum increases in plant fresh weight were recorded with *S.nobilis* WA-3 (84%) and *S. djakartensis* TB-4 (75%), increased plant dry weight was recorded in case of *S. nobilis* WA-3(85%), *S. djakartensis* TB-4 (66%), and *S enissocaesilis* TA-3 (60%). In case of number of leaves, significant increases were recorded with *S. nobilis* WA-3 (27%), *S. djakartensis* TB-4 (27%), and *S. enissocaesilis* TA-3 (23%). Significant increase in case of number of roots were recorded in case of *S. nobilis* WA-3 (30%), *S. djakartensis* TB-4 (26%), and *S. enissocaesilis* TA-3 (22%) as compared to water treated control (Figure 5).

**Table 4:** **Effect of selected actinomycetes on the growth of Wheat (*Triticum aestivum*)**.

**Isolates**	**Growth parameters of actinomycetes treated wheat plants**					
	**Root length (cm)**	**Shoot length (cm)**	**No. of leaves**	**No. of roots**	**Seedling fresh weight (g)**	**Seedling dry weight (g)**
Control	13.1 ± 1.4a	116.2 ± 0.7 a	3.13 ± 0.1 a	4.3 ± 0.6 a	11.4 ± 0.7 a	2.32 ± 0.1a
WA-1	15.0 ± 10.9ab	21.7 ± 1.1b	3.4 ± 0.1ab	4.6 ± 0.3ab	13.9 ± 0.6bc	2.81 ± 0.2b
WA-3	23.8 ± 1.2f	26.8 ± 0.3c	4 ± 0d	5.6 ± 0.2b	21.0 ± 0.5d	4.33 ± 0.1d
WC-3	16.0 ± 0.6bc	22.0 ± 0.7b	3.46 ± 0.1ab	4.93 ± 0.2ab	14.3 ± 0.4b	3.08 ± 0.1b
WD-3	18.4 ± 0.4cd	22.4 ± 1.0b	3.53 ± 0.1bc	5 ± 0.4ab	15.5 ± 0.7b	3.17 ± 0.1b
TA-3	19.9 ± 0.4de	24.8 ± 1.0c	3.86 ± 0.09cd	5.26 ± 0.4ab	17.4 ± 0.5c	3.72 ± 0.06c
TB-4	22.2 ± 0.6ef	25.0 ± 0.5c	4 ± 0d	5.46 ± 0.1ab	20.0 ± 0.5d	3.87 ± 0.2cd

Control, non-inoculated. Data is the mean of 15 plants obtained from three replicates. ± Symbol indicate values for standard error of means. Different letters indicate significant difference between treatments, using Duncan's multiple range test (P = 0.05).

### Identification of selected actinomycetes strains by 16s rRNA gene sequencing

**Figure 6:**
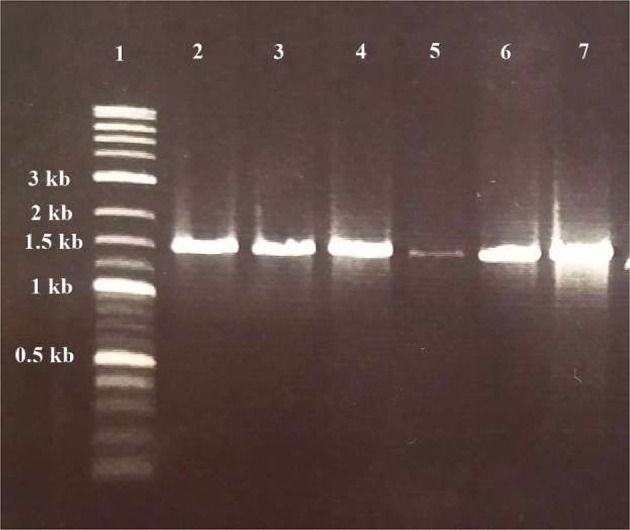
**Gel electrophoresis of PCR products for detection of Actinomycetes isolates**. Lane 1: DNA ladder (Fermentas, Germany); lane 2, 3, 4, 5, 6, and 7: positive samples of *Streptomyces* sp. (WA-1), *S. nobilis* (WA-3), *S. kunmingenesis* (WC-3), *S. mutabilis* (WD-3), *Streptomyces enissocaesilis* (TA-3), *and S. djakartensis* (TB-4) respectively (1.5 kb).

Six of them including WA-1, WA-3, WC-3, WD-3, TA-3, and TB-4 were selected based on their ability to produce phytohormone IAA, solubilization of inorganic phosphates, ACC deaminase activity, siderophore, ammonia, and hydrogen cyanide production. For all the selected actinomycetes, single band PCR product of 1.5 kb length were achieved with the universal primers (Figure 6). The sequences of 16S rRNA gene were analyzed by comparison with sequences in GenBank through Nucleotide BLAST (http://www.ncbi.nlm.nih.gov/BLAST). After comparison, strains WA-1 showed 98% similarity with *Streptomyces* sp. while the strains WA-3, WC-3, WD-3, TA-3, and TB-4 showed 99% similarity with *Streptomyces nobilis, Streptomyces kunmingenesis, Streptomyces mutabilis, Streptomyces enissocaesilis, Streptomyces djakartensis*, respectively. The sequences from strains WA-1, WA-3, WC-3, WD-3, TA-3, and TB-4 have been deposited in the GenBank and accession numbers were obtained (Table 5).

**Table 5:** **Genetic similarity of the selected strains with different species of the genus Streptomyces determined by 16S rRNA gene sequencing**.

**Strains**	**Source of isolation, Plant**	**Identified as**	**Similarity (%)**	**Accessions**
WA-1	Rhizophere soil, *Triticum aestivum*	*Streptomyces* sp.	98	KU750781
WA-3	Rhizophere soil, *Triticum aestivum*	*Streptomyces nobilis*	99	KU750782
WC-3	Rhizophere soil, *Triticum aestivum*	*Streptomyces kunmingensis*	99	KU750783
WD-3	Rhizophere soil, *Triticum aestivum*	*Streptomyces mutabilis*	99	KU750784
TA-3	Rhizopshere soil, *Solanum lycopersicum*	*Streptomyces enissocaesilis*	99	KU750780
TB-4	Rhizopshere soil, *Solanum lycopersicum*	*Streptomyces djakartensis*	99	KU750779

## Discussion

In this study, a total of 98 actinomycetes strains were isolated from wheat and tomato rhizospheric soil. All of the isolates were screened for different PGP traits and 30 of them were found to be showing excellent PGP activities in initial screening. Out of these, six most promising actinobacterial strains belonging to the genus *Streptomyces* were investigated by *in-vivo* studies. The 16S rRNA gene sequence of these six selected actinomycetes strains including WA-1, WA-3, WC-3, WD-3, TA-3, and TB-4 showed maximum sequence similarity with members of the genus *Streptomyces.*

Majority of the PGPR actinomycetes synthesize IAA which is responsible for increased number of adventitious roots which help plant to uptake a large volume of nutrients and absorb water, while increased root exudates in turn benefits the bacteria (El-Tarabily, 2008). In our study, the IAA production ranges between 10 and 79.5 μg/ml and *Streptomyces nobilis* strain WA-3 was detected as the best strain for the IAA production (79.5 ug/ml) that exceeds the level of previously reported work by Khamna et al. (2009). Abd-Alla et al. (2013) reported *Streptomyces* sp. CMU-MH021 that could produce 28.5 μg/ml of IAA. Several *Streptomyces* species, such as *S. olivaceoviridis, S. rimosus, S. Rochei, S. griseoviridis, and S. lydicus* have the ability to produce IAA and improve plant growth by increasing seed germination, root elongation and root dry weight (Mahadevan and Crawford, 1997).

Soil acidification often resulted due to the growth of phosphate-solubilizing bacteria (PSB), which in turn resulted in phosphorus solubilization. Therefore, PSB are well known as solubilizers of inorganic phosphate (Verma et al., 2001). The maximum phosphate solubilization activity was detected in the strain *Streptomyces* sp. WA-1 (72.13 mg/100 ml). Hamdali et al. (2008) reported 83.3, 58.9, and 39 mg/100 ml phosphate solubilization by *Streptomyces cavourensis, Streptomyces griseus*, and *Micromonospora aurantiaca*, respectively.

Almost all of the rhizospheric actinomycetes were also able to produce ammonia and hydrogen cyanide. Marques et al. (2010) recommended that bacteria can synthesize ammonia and supply nitrogen to the host plant. Additionally, over production of ammonia serve as a prompting factor for the virulence of opportunistic plant pathogens. Ammonia production also plays an important role HCN production play an essential role in suppression of plant disease. In this study, all the ammonia and HCN producing isolates belong to the genus *Streptmyces.* Similarly, Husen et al. (2011) reported *Streptomyces* sp. LSW05 strain as a potent HCN producer.

Siderophore production is one more feature that stimulates plant growth by forming complex with iron form (Fe 3^+^) in the rhizosphere making iron unavailable to the phytopathogens. It is suggested by Tan et al. (2009) siderophore production that the production of siderophore is an important factor for phytopathogen antagonism and developing growth of the plant. In our study, we detected the 85.7% isolates positive for siderophore production. Similarly, Khamna et al. (2010) has revealed that *Streptomyces* CMU-SK 126 isolated from Curcuma mangga rhizospheric soil exhibited high amount of siderophore.

ACC deaminase-producing bacteria have been known to promote plant growth by decreasing ethylene inhibition of various plant processes (Husen et al., 2011). They can increase root growth by lowering endogenous ACC levels (Glick, 2005). Plant roots must be able to perceive and recognize such elicitors in ways similar to the recognition of elicitors from plant pathogens. In fact, plant pathogens might interfere with the action of PGPR by being perceived by similar receptors (Husen, 2003).

The effect of soil microbes on PGP including root development has been reported by Uphoff et al. (2009). In the present study, root elongation assay and pot experiment performed by using wheat seeds inoculated with PGP *Streptomyces* strains, significantly enhanced the plant growth by increasing plant root length, plant shoot length, dry weight, fresh weight, number of leaves, and number of roots over the un-inoculated control. The *Streptomyces* strains are extensively reported in the literature for its PGP potential (Nassar et al., 2003; El-Tarabily, 2008; Gopalakrishnan et al., 2011b). As hypothesized earlier, the mechanism by which the *Streptomyces* enhanced the morphological and yield parameters on both sorghum and rice could be their PGP traits including IAA and siderophore production (direct stimulation of PGP; Gopalakrishnan et al., 2011b).

## Conclusion

The study revealed that these rhizospheric actinomycetes are potential microbial inoculants because of their intensified PGP activities such as IAA production, phosphate solubilization, siderophore, and HCN production and ACC deaminase production. The strains reported in this study are promising candidates to be developed as commercial biofertilizer formulation and can also be exploited for the production of various agroactive compounds like auxins etc. As such this is the first comprehensive report from Pakistan about the PGP traits and potential agricultural applications of actinomycetes.

## Author contributions

SA: Isolation, biochemical characterization, identification and *in-vitro* and *in-vivo* screening of actinomycetes for multiple PGP traits/hormones. Did all the experimental work. IS: Supervise in the sampling of the rhizospheric soils. Teach how to isolate actinomycetes by using enrichment, help in DNA isolation and PCR purification technique. BA: Co-supervise in performing PGP experiments. Both *in-vitro* and *in-vivo* screening of actinomycetes.

### Conflict of interest statement

The authors declare that the research was conducted in the absence of any commercial or financial relationships that could be construed as a potential conflict of interest.
