# Supplementary material for: Screening of Rhizospheric Actinomycetes for Various In-vitro and In-vivo Plant Growth Promoting (PGP) Traits and for Agroactive Compounds
Source: Front Microbiol. 2016 Aug 29;7:1334. doi: 10.3389/fmicb.2016.01334 (PMC5002418; doi:10.3389/fmicb.2016.01334)
Supplement: Supplementary file 1 [file DataSheet1.DOCX]

**Screening of rhizospheric actinomycetes for various *in-vitro* and *in-vivo* plant growth promoting (PGP) traits and for agroactive compounds**

Sumaira Anwar^1^, Basharat Ali^1^, Imran Sajid^1*^

^1^Department of Microbiology and Molecular Genetics, University of the Punjab, Lahore, Punjab, Pakistan

*** Correspondence:**Imran Sajid
[imran.mmg@pu.edu.pk](mailto:imran.mmg@pu.edu.pk)


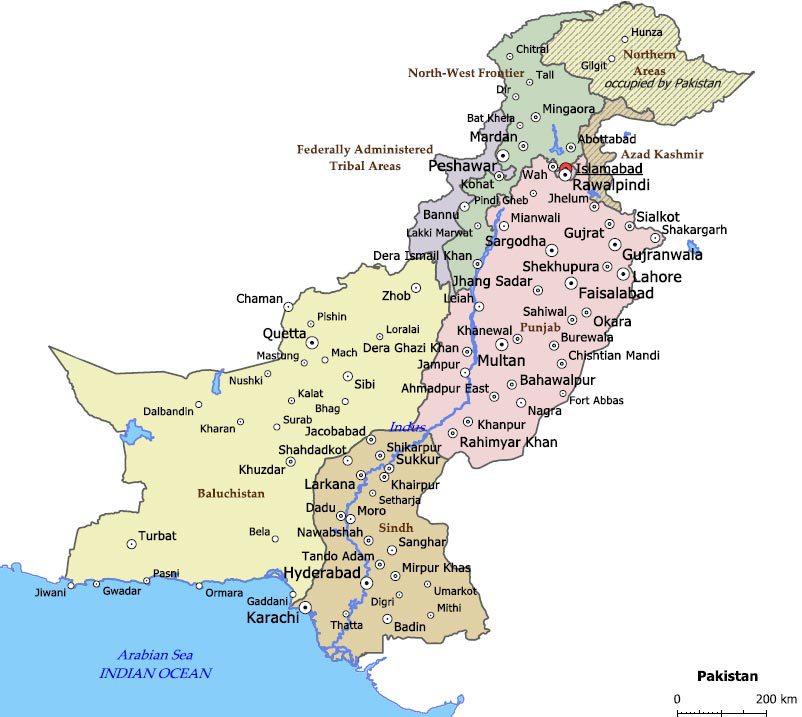


**Figure S1:** Map showing the isolation sites for the rhizospheric actinomycetes strains investigated in the study (arrows indicates the three major cities, Lahore, Gujranwala, sheikhupura)

**Table S1:** Morphological characteristics of the selected 30 rhizosperic actinomycetes

| **Actinomycetes**  **Strains** | **Colony diameter (cm)** | **Color of aerial mycelium** | **Color of substrate mycelium** | **Identified as** |
| --- | --- | --- | --- | --- |
| WA-1 | 0.6 | green | Beige yellow | *Streptomyces* sp. |
| WA-2 | 0.2 | grass green | Yellow | *Streptomyces* sp. |
| WA-3 | 0.3 | orange | Orange red | *Streptomyces* sp. |
| WA-4 | 0.5 | Dark grey | Ivory | *Streptomyces* sp. |
| WA-5 | 0.3 | Salmon orange | Red orange | *Streptomyces* sp. |
| WB-1 | 0.8 | Papyrus white | Brown beige | *Streptomyces* sp. |
| WB-2 | 0.4 | Chocolate brown | Oyster white | *Streptomyces* sp. |
| WB-3 | 0.4 | Moss grey | Green | *Streptomyces* sp. |
| WC-1 | 0.8 | Cream | Ivory | *Streptomyces* sp. |
| WC-2 | 0.3 | Light grey | Ochre brown | *Streptomyces* sp. |
| WC-3 | 0.3 | light yellow | Olive yellow | *Streptomyces* sp. |
| WD-1 | 0.3 | Cream | Brown beige | *Streptomyces* sp. |
| WD-2 | 0.3 | Cream | Ivory | *Streptomyces* sp. |
| WD-3 | 0.4 | Pale yellow | Light brown | *Streptomyces* sp. |
| WD-4 | 0.3 | Chrome green | Olive grey | *Streptomyces* sp. |
| TA-1 | 0.5 | Light grey | quarts grey | *Streptomyces* sp. |
| TA-2 | 0.3 | Silver grey | Tarpaulin grey | *Streptomyces* sp. |
| TA-3 | 0.5 | Platinum grey | Clay brown | *Streptomyces* sp. |
| TB-1 | 0.1 | Concrete grey | Grey beige | *Streptomyces* sp. |
| TB-2 | 0.5 | Grey white | Broom yellow | *Streptomyces* sp. |
| TB-3 | 0.8 | Silver grey | Clay brown | *Streptomyces* sp. |
| TB-4 | 0.2 | Dusty grey | Clay brown | *Streptomyces* sp. |
| TC-1 | 0.5 | Light grey | Beige brown | *Streptomyces* sp. |
| TC-2 | 0.4 | Bright red orange | Orange | *Streptomyces* sp. |
| TC-3 | 0.5 | Pastel green | Olive green | *Streptomyces* sp. |
| GA-1 | 0.4 | Silver grey | Brown beige | *Streptomyces* sp. |
| GA-2 | 0.5 | Pigeon blue | Green brown | *Streptomyces* sp. |
| GA-3 | 0.6 | Dark grey | Clay brown | *Streptomyces* sp. |
| BA-1 | 0.7 | Dark grey | Grey beige | *Streptomyces* sp. |
| BA-2 | 0.4 | Pastel green | Reseda green | *Streptomyces* sp. |

**Table S2:** Biochemical and physiological characteristics of the selected 30 rhizosperic actinomycetes

| Actinomycetes strains | Carbon source utilization | | | | | | | Hydrolysis of | | | | |
| --- | --- | --- | --- | --- | --- | --- | --- | --- | --- | --- | --- | --- |
|  | Glu | Xyl | Suc | Man | Ara | Mann | Raf | Tyr | Xan | Hypo-  xan | Starch | Urea |
| WA-1 | + | - | - | + | + | + | + | + | + | + | + | + |
| WA-2 | + | + | + | + | + | - | + | - | + | - | + | + |
| WA-3 | + | - | + | + | + | + | + | + | - | + | + | + |
| WA-4 | + | + | + | + | + | - | + | + | + | + | + | + |
| WA-5 | + | + | + | - | + | - | - | + | + | - | + | + |
| WB-1 | + | + | + | + | + | - | + | - | + | + | + | + |
| WB-2 | + | + | + | + | + | - | + | + | + | + | + | + |
| WB-3 | + | + | + | - | + | - | + | + | - | + | + | + |
| WC-1 | + | + | + | - | + | - | + | + | + | + | + | + |
| WC-2 | + | + | - | + | + | + | + | + | + | + | + | + |
| WC-3 | + | - | + | + | - | + | - | - | + | + | + | + |
| WD-1 | + | + | + | + | + | - | + | - | + | + | + | + |
| WD-2 | + | + | + | + | + | + | + | - | + | + | + | + |
| WD-3 | + | + | + | + | + | + | - | + | + | + | + | + |
| WD-4 | + | + | + | + | + | + | + | - | - | + | + | + |
| TA-1 | + | + | + | + | + | + | + | + | + | + | + | + |
| TA-2 | + | + | - | + | + | - | + | + | + | + | + | + |
| TA-3 | + | + | - | + | + | + | + | - | - | - | + | + |
| TB-1 | + | + | + | + | + | - | + | - | - | + | + | + |
| TB-2 | + | + | - | + | + | - | + | + | + | + | + | + |
| TB-3 | + | + | + | - | + | - | - | + | + | + | + | - |
| TB-4 | + | + | + | - | - | + | + | + | + | + | + | + |
| TC-1 | + | + | - | + | - | - | + | + | + | - | + | - |
| TC-2 | + | + | + | - | - | + | + | + | + | - | + | + |
| TC-3 | + | + | + | + | + | + | + | + | + | + | + | + |
| GA-1 | + | + | + | + | - | - | + | + | + | + | + | + |
| GA-2 | + | + | + | + | + | - | + | - | + | + | + | + |
| GA-3 | + | + | + | + | + | - | + | + | + | + | + | + |
| BA-1 | + | + | + | + | + | - | + | + | + | + | + | + |
| BA-2 | + | + | + | - | + | - | - | + | + | + | + | + |

**+=growth, -=no growth** (Glu=D-glucose, Xyl=D-xylose, Suc=sucrose, Man=D-mannose, Ara=L-arabinose, Mann=D-mannitol, Raf=D-raffinose, Tyr=L-tyrosine, Xan= Xanthine, Hypo-xan= Hypo-xanthine)s


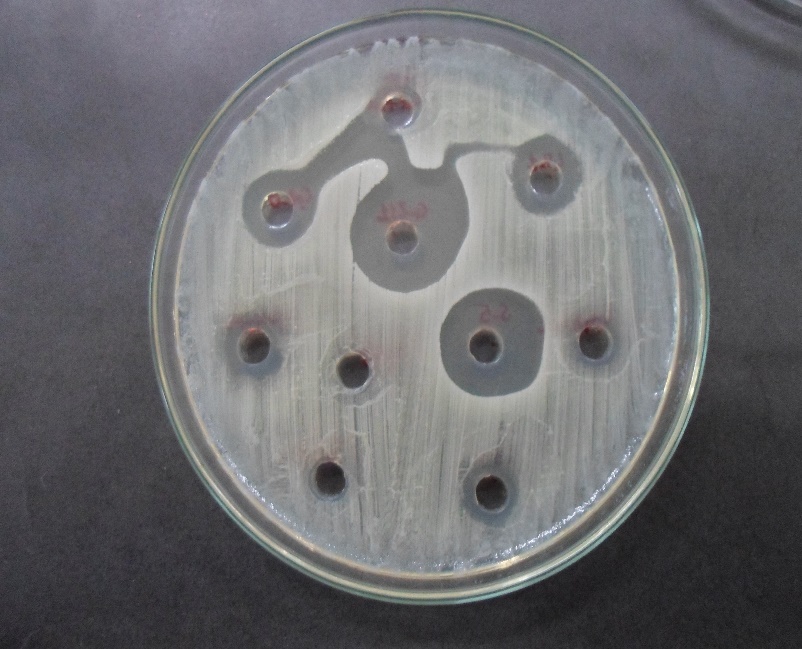

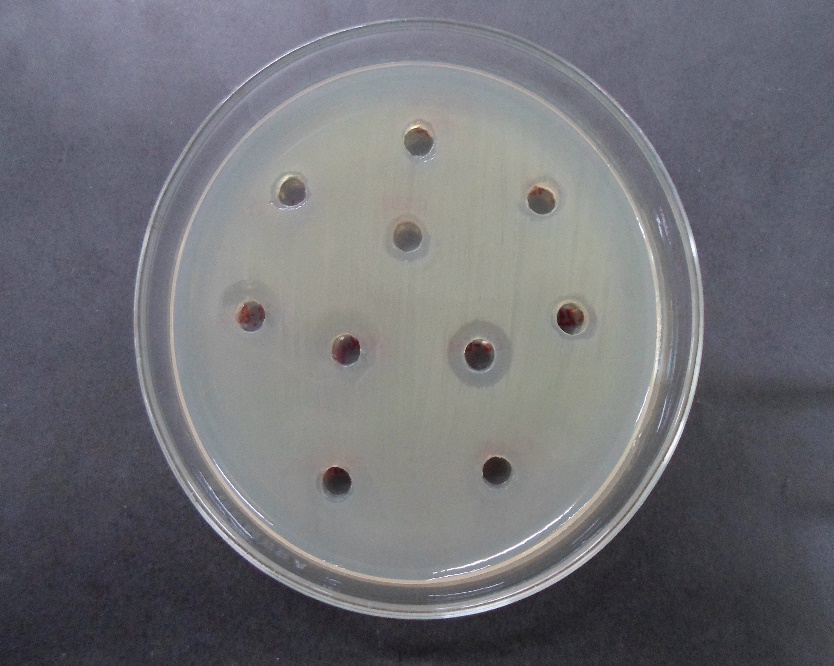


A) B)

**Figure S2:** Antagonistic potential of methanolic extract (60 µl in each well) of the selected rhizospheric actinomycetes against A) *Bacillus* B) *Pseudomonas* by using agar diffusion method
